# The Value of Voltage Histogram Analysis Derived Right Atrial Scar Burden in the Prediction of Left Atrial Scar Burden

**DOI:** 10.1155/2020/3981684

**Published:** 2020-08-13

**Authors:** Szilvia Herczeg, Joseph Galvin, John J. Keaney, Edward Keelan, Roger Byrne, Claire Howard, Laszlo Geller, Gabor Szeplaki

**Affiliations:** ^1^Heart and Vascular Centre of Semmelweis University, 68 Varosmajor Street, 1122 Budapest, Hungary; ^2^Atrial Fibrillation Institute, Mater Private Hospital, Eccles Street, Dublin 7, Ireland; ^3^Heart and Vascular Centre, Mater Private Hospital, Eccles Street, Dublin 7, Ireland

## Abstract

**Introduction:**

Growing evidence suggests that fibrotic changes can be observed in atrial fibrillation (AF) in both atria. Quantification of the scar burden during electroanatomical mapping might have important therapeutic and prognostic consequences. However, as the current invasive treatment of AF is focused on the left atrium (LA), the role of the right atrium (RA) is less well understood. We aimed to characterize the clinical determinates of the RA low-voltage burden and its relation to the LA scaring.

**Methods:**

We have included 36 patients who underwent catheter ablation for AF in a prospective observational study. In addition to LA mapping and ablation, high-density RA bipolar voltage maps (HD-EAM) were also reconstructed. The extent of the diseased RA tissue (≤0.5 mV) was quantified using the voltage histogram analysis tool (CARTO^®^3, Biosense Webster).

**Results:**

The percentage of RA diseased tissue burden was significantly higher in patients with a CHA_2_DS_2_-VASc score ≥ 2 (*p* = 0.0305), higher indexed LA volume on the CTA scan and on the HD‐EAM (*p* = 0.0223 and *p* = 0.0064, respectively), or higher indexed RA volume on the HD‐EAM (*p* = 0.0026). High RA diseased tissue burden predicted the presence of high LA diseased tissue burden (OR = 7.1, CI (95%): 1.3–38.9, *p* = 0.0145), and there was a significant correlation of the same (*r* = 0.6461, *p* < 0.0001).

**Conclusions:**

Determining the extent of the right atrial low-voltage burden might give useful clinical information. According to our results, the diseased tissue burden correlates well between the two atria: the right atrium mirrors the left atrium.

## 1. Introduction

There is remarkable scientific and clinical interest in identifying the cause of atrial fibrillation (AF) and the predictors of AF recurrence after rhythm control therapy. Among these factors, atrial fibrosis is one of the most challenging mechanisms to understand [1–4]. The burden of left atrial (LA) fibrosis can be estimated by cardiac MRI or by invasive endocardial bipolar voltage mapping defining fibrosis as a low-voltage area (LVA) [5–9].

Recently, we have described that voltage histogram analysis (VHA), a novel tool used in electroanatomical mapping, was able to assess the burden of LA LVA [10]. There is growing evidence suggesting that more severe LA fibrosis is detected in the elderly and in AF patients with comorbidities such as hypertension, heart failure, diabetes, or obesity [3, 9, 11, 12]. The fibrotic LA area is also more extensive if larger LA with/or impaired atrial function is detected [5]. However, fibrotic atria can also be identified in patients with lone AF [13]. Thus, determining the predictive value of the extent of LA fibrosis would be important for an electrophysiologist and might lead to a more personalised treatment of a patient with AF undergoing catheter ablation.

Although AF is known to cause biatrial fibrosis, data in the literature focused on the analysis of the right atrial (RA) LVA assessed during electroanatomical mapping are scarce. Our aim was to determine the value of RA fibrosis in the prediction of LA fibrosis and to identify its predisposing factors. We hypothesised that the fibrotic burden of the RA might mirror the extent of the LA LVA and correlate to its severity. As determining the extent of the low-voltage areas can be time consuming, we have aimed to use the novel VHA tool, which allows rapid, accurate and quantitative assessment of LVA during electroanatomical mapping of the heart.

## 2. Methods

### 2.1. Study Population

Patients with nonvalvular symptomatic, antiarrhythmic drug-refractory paroxysmal or persistent atrial fibrillation who underwent a pulmonary vein isolation (PVI) procedure at the Mater Private Hospital, Dublin, in 2017 were consecutively included in a prospective, single-centre observational study. The results of the LA VHA and its relation to the AF risk factors have been previously published [10]. In the present study, we have included a subset of the patients from the original cohort where additional right atrial mapping was also performed. Patients were excluded if they had any previous right or left atrial ablation in their medical history or if sinus rhythm could be not maintained during mapping despite electrical cardioversion attempts. All patients who were enrolled to the study gave informed consent. The study protocol was reviewed and approved by the Mater Misericordiae University Hospital Clinical Research Ethics Committee and was in accordance with the Declaration of Helsinki.

### 2.2. Measurement of Atrial Diameters and Volumes

As a standard, all patients underwent a preprocedural computed tomography angiography (CTA) scan of the left atrium and right atrium using a SOMATOM Definition Dual Source CT scanner (Siemens, Munich, Germany). The shortest longitudinal atrial diameters (L) and end-systolic atrial area were measured on two- (A1) and four-chamber (A2) views of the CTA images using the Carestream version 11 software (Phillips, Amsterdam, The Netherlands). LA volume and RA volume (LAV/RAV ml) were calculated according to the following area-length method based on the literature (8/3*π x* [(A1) *x* (A2)/L]) [14–16]. Afterwards, the indexed LAV and RAV (LAVI/RAVI, ml/m^2^) were calculated based on the body surface area (BSA) of each patient. In addition, atrial volumes were also determined by the CARTO^®^ 3 electroanatomical mapping system (Biosense Webster Inc., Diamond Bar, California, USA), based on the maps acquired by the fast anatomical mapping (FAM).

### 2.3. Biatrial Voltage Mapping and Catheter Ablation

The details of the LA mapping and the PVI were previously described [10]. Ablation procedures were performed under general anaesthesia with the guidance of transoesophageal echocardiography and fluoroscopy. If the patient was in atrial fibrillation, electrical cardioversion was performed prior to the mapping. After a double transseptal puncture, first, the LA was mapped; next, PVI was performed, and then, the multielectrode mapping catheter was withdrawn to the RA and a bipolar voltage map was acquired.

Anatomical and voltage mapping of the atria were performed by using a circular 20-pole Lasso catheter (Biosense Webster) during sinus rhythm or with pacing from the proximal coronary sinus electrode at 600 ms in case of hemodynamically significant sinus bradycardia or junctional rhythm related to the general anaesthesia. The CONFIDENSE™ and FAM modules of the CARTO^®^3 mapping system were used with the tissue proximity indication filter turned on. In order to create an accurate and high-density map with even distribution, we were aiming to acquire at least 500 points per map. In case of difficult anatomy, additional points were collected with a single-tip irrigated contact force sensing ablation catheter (ThermoCool SmartTouch™ 8Fr, Biosense Webster). All points containing ventricular far-field, noise, and pacing spike artefact were excluded automatically and manually as well.

The high-density electroanatomic map (HD-EAM) was merged with the preprocedurally segmented CTA image for ablation guidance. Pulmonary vein isolation was performed by radiofrequency energy with a bilateral point-by-point wide antral circumferential ablation with the ablation catheter inserted through a steerable sheath (Agilis, Abbott Inc.). In case of documented typical atrial flutter, an additional cavotricuspid isthmus line procedure was performed after RA mapping.

### 2.4. Voltage Map Analysis

HD-EAMs were analysed offline after the procedure using a novel voltage area analysis software algorithm, the CARTO^®^3 VHA tool (Biosense Webster). The detailed methodology of the analysis was described in our previous publication [10]. In each case, the LA appendage, the pulmonary veins, and the mitral annulus were manually excluded from the LA map, while the inferior and superior vena cava and the tricuspid annulus were excluded from the RA map.

**Figure 1:**
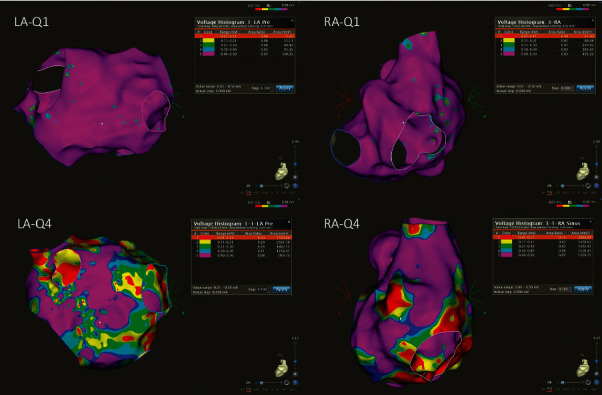
High-density electroanatomic bipolar voltage maps of two representative patients (posteroanterior (PA) view). Patient 1, an example with low amount of the low-voltage area (Quartile 1, Q1) in the left atrium (LA) and right atrium (RA). Patient 2, an example with Quartile 4 (Q4) diseased tissue of the left and the right atrium.

We defined the diseased tissue as bipolar voltage ≤0.5 mV. The burden of the diseased LA tissue and RA tissue was measured by using the VHA tool and is reported as percentages of the whole mapped LA and RA, respectively. We created quartiles of the diseased RA and LA volumes in order to categorize patients into Q1 (lowest quartile of diseased tissue) to Q4 (highest quartile of diseased tissue), respectively (RA-Q1-4; LA-Q1-4, Figure 1.).

### 2.5. Statistical Analysis

Statistical analysis was performed by Prism version 6.01 (GraphPad Software, Inc., La Jolla, CA). Normality was assessed by the Shapiro–Wilk test. As most variables had nonparametric distributions, the Mann–Whitney *U* test was used to compare continuous variables, and results are reported as medians and interquartile ranges. Categorical variables were compared using the chi-square test and reported as absolute numbers (frequencies). Spearman's correlation analysis was performed to assess the correlation of the LA and RA parameters. Furthermore, the Kruskal–Wallis test was conducted for multiple comparisons. A two-tailed *p* < 0.05 was considered as significant.

## 3. Results

### 3.1. Patient Characteristics

We included 36 patients in the final analysis, with a median age of 69 (58–75) years, mainly male (75%), and presenting with persistent AF in 58% (Table 1). The studied population had a CHA_2_DS_2_-VASc score of two or higher in 64%. Hypertension was present in 42%, coronary artery disease in 22%, diabetes mellitus in 11%, and prior stroke or TIA in 8% in our cohort. Nine patients had structural heart disease (7 with ischaemic heart disease, 1 with non-ischaemic dilated cardiomyopathy, and 1 with prior aortic valve replacement and coronary bypass graft surgery). The median of the left ventricular ejection fraction (LVEF) was in the normal range (56% (52–61%)). Left atrial and right atrial indexed volumes were mildly elevated on the CTA images with the median of 59 (50–78) ml/m^2^ and 53 (37–66) ml/m^2^, respectively.

### 3.2. Biatrial Mapping and Voltage Analysis

During the procedure, nearly a median of one thousand homogenous points were collected for LA and RA in a median of 8 and 7 minutes, respectively (Table 2). The maps were taken predominantly in sinus rhythm, with the exception of 2 cases, where pacing from the proximal coronary sinus electrode was required to maintain hemodynamic stability. The median diseased tissue burden (≤0.5 mV) assessed by using the VHA tool was 19 (13–53) % for LA and 24 (14–34) % for RA. The diseased LA tissue burden showed similar relations to the baseline characteristics, as previously published (data not shown) [10]. We focused on determining the relationship between the baseline characteristics and the median percentage of the diseased RA tissue burden. Patients with a CHA_2_DS_2_-VASc score ≥2 (*p* = 0.0305), larger LAVI measured on CTA (*p* = 0.0223) and on HD-EAM (*p* = 0.0064), and larger RAVI measured on HD-EAM (*p* = 0.0026) had a significantly higher RA diseased tissue burden (Table 3, the Mann–Whitney *U* test).

### 3.3. Correlation between the RA and LA Diseased Tissues

First, we have analysed the correlation of LA and RA low-voltage area percentages in the whole spectrum (0.1–0.5 mV, with 0.1 mV steps), and we found that there was a strong correlation by using all cutoffs (≤0.1 mV: *p* < 0.0001, *R* = 0.5760, ≤0.2 mV: *p* < 0.0001, *R* = 0.6250, ≤0.3 mV: *p* < 0.0001, *R* = 0.6120, ≤0.4 mV: *p* < 0.0001, *R* = 0.6660, and ≤0.5 mV: *p* < 0.0001, *R* = 0.6461, Spearman's correlation). Next, we determined whether or not the RA parameters would relate to the diseased LA tissue burden where the diseased tissue was defined as a bipolar voltage of ≤0.5 mV in accordance with our previous publication [10]. Patients were divided into 2 subgroups according to their percentage of the LA diseased tissue: patients with the highest burden (in the upper quartile of the diseased LA tissue, LA-Q4) formed one group and those in the lower quartiles (LA-Q1-3) formed the other.

**Figure 2:**
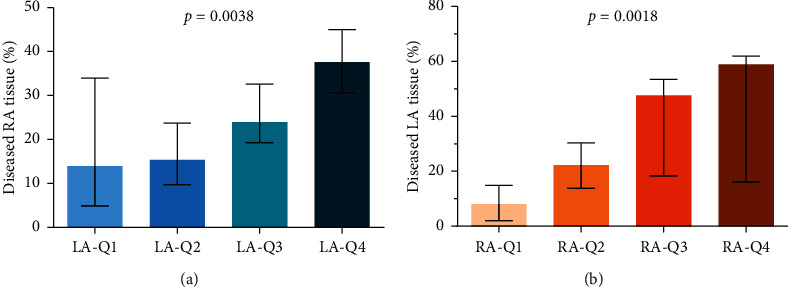
Comparison of left atrial (LA) and right atrial (RA) diseased tissue burden (Kruskal–Wallis test, the error bars representing median and interquartile ranges; Q-quartiles).

Patients with higher RA LVA quartiles had higher percentage of the LA diseased tissue (*p* = 0.0038), while patients with higher LA LVA quartiles had higher percentage of the RA diseased tissue (*p* = 0.0018, Figure 2, Kruskal–Wallis test). High diseased RA tissue burden (RA-Q4) predicted the extent of diseased LA tissue: patients in RA-Q4 significantly more frequently belonged to LA-Q4 (OR = 7.1, CI (95%): 1.3–38.9, *p* = 0.0145, chi-square test).

## 4. Discussion

In the present study, we have described that RA diseased tissue burden can rapidly be quantified by the novel VHA tool during high-density electroanatomical mapping of the heart. The extent of the RA low-voltage area did not show any correlation with individual risk comorbidities of AF but did with the CHA_2_DS_2_-VASc score. Patients with a CHA_2_DS_2_-VASc score ≥2 had significantly higher RA diseased tissue burden. Higher LA and RA volume also correlated with high RA diseased tissue burden. The presence of high percentage of low-voltage areas in the RA predicted the same in the LA, and there was a strong correlation of the percentage of diseased tissue burden between the two atria.

The exact cause of the development and maintenance of atrial fibrillation is still an unsolved phenomenon. The remodelling is characterised by activation of fibroblasts, development of atrial fibrosis and inflammatory infiltration, causing local heterogeneity in conduction leading to the rise of a micro-reentrant arrhythmia [17, 18]. The known risk factors of AF contribute to the structural remodelling of the atrial tissue and consistent loss of atrial function [1, 19]. Moreover, the increasing atrial pressure followed by atrial dilatation plays an important role in the pathophysiology as well [20]. Interestingly, those mechanisms are also observed in lone AF, in the absence of comorbidities. Kottkamp et al. suggested that the presence of the fibrotically destroyed atrial tissue cannot be solely explained by ageing or an underlying heart disease or the AF burden itself, but AF is an element of the independent “arrhythmic manifestation” of the progressing fibrotic atrial cardiomyopathy [13].

It is also still a burning question how to precisely quantify diseased atrial tissue burden. Various methods have been developed to determine the extent of atrial fibrosis, either using cardiac imaging (e.g. MRI) or by analysing the intracardiac electrograms during HD electroanatomical mapping (based on conduction velocity, complex fractionated electrograms, or voltage mapping). Cardiac MRI is a useful tool to detect late gadolinium enhancement (LGE) areas, which would indicate the presence of fibrosis in the atria. However, variances in the imaging protocols, the subsequent difficulties with reproducibility, and the relatively low resolution (considering the aim is to determine the fibrotic burden of the atrial tissue, which has only a few millimetres thickness) limit the widespread use of this technology in the routine clinical practice [21]. On the other hand, analysis of HD electroanatomical maps is widely available, and the resolution has significantly increased by the evolution of the high-density mapping tools. Voltage mapping allows us to detect the exact voltage values gathered from the atrial tissue with special resolution, which has a precision within millimetres. However, point-by-point analysis of the signals might be time consuming, considering the high number of points collected during the mapping. Visual estimation of low-voltage areas has been shown to overestimate the amount of dense scar and underestimate the extent of diseased atrial tissue areas [22]. To overcome these limitations, we have described that an automated software analysis, the VHA tool (CARTO^®^3, Biosense Webster), is able to analyse thousands of points within negligible time and is able to determine the exact percentage of the low-voltage areas relating to the whole surface [10]. The VHA tool has proven useful in rapid determination of LA scar burden, which has shown good correlation to known AF risk factors [10].

The vast majority of studies is focused on left atrial remodelling, and only few have demonstrated that the right atrium is also effected in AF patients. Akutsu et al. concluded in a review that occurrence of AF causes symmetrical structural and electric changes in both atria, and the biatrial remodelling independently affect the success of ablation for AF [23]. Frustaci et al. analysed right atrial biopsy samples of patients with lone atrial fibrillation, which showed abnormal histological finding, which was absent in healthy controls [24]. Xu et al. confirmed that fibrotic changes can be also detected at the molecular level in the RA [25]. These studies implicate that the fibrotic changes at cellular and subcellular levels do happen in both atria, and they are not limited to the LA.

Biatrial remodelling was also detected by both MRI and voltage analysis [26]. Prabhu et al. has also shown that the burden of biatrial fibrosis is higher in patients with reduced left ventricular ejection fraction (LA: 23% vs. 6%; RA: 20% vs. 11%) [27]. Our data on biatrial low-voltage areas show a clear relation between the two atria as well. Patients with greater LA low-voltage burden presented with higher percentage of low-voltage areas in their RA, and vice versa, and patients with higher RA low-voltage burden presented with a higher extent of low-voltage areas in their LA. A strong correlation was found between the extent of the LA and RA diseased tissues. Prabhu et al. performed biatrial mapping in 40 persistent AF patients, and tissue voltage, complex electrograms, and conduction velocity were also measured [28]. Consistent with our results, they have shown that the LA and RA bipolar voltages showed a good correlation (*R* = 0.57, *p* < 0.001), and there was no significant difference between their global bipolar voltage (LA 1.89 ± 0.77 mV vs. RA 1.77 ± 0.57 mV, *p*=0.57).

In their study, multielectrode mapping was not used, resulting in a lower point density (LA mean 220 points and RA mean 216 points are compared to LA median 987 points and RA median 897 points in our study). Moreover, they have determined the percentage of low voltage by directly calculating the number of low-voltage points related to the number of all collected points but not the actual surface. They have also shown that there is a correlation between fractionated signals and conduction velocity between the two atria, fractionated signals within low-voltage areas also correlate between, and a remarkable amount of fractionation is related to the low voltage areas [28]. However, point-by-point analysis of fractionated signals or determining conduction velocity can be time consuming.

The main aim of this study was to present a method, which allows rapid automated voltage analysis and to confirm that by using this method, the LA LVA can be predicted by RA LVA analysis. The novel VHA tool we used for our analysis creates a mesh around the mapped surface first and calculates an average voltage value for every subarea in the total mapped atrial surface defined by the triangulated mesh. This results in a more accurate determination of the exact percentage of low-voltage areas and better estimation of the scar burden. The automated analysis offers a rapid calculation, in contrast with the manual method Prabhu et al. were describing. Therefore, our present results reenforce those of Prabhu et al.'s and further extends it, by suggesting a reliable alternative method for the RA voltage analysis [28].

The first main clinical impact of our finding is the description of an automated voltage analysis tool, which allows rapid LVA quantification in the RA. Second, we confirmed that valuable information can be gathered by determining RA diseased tissue burden in an AF patient who undergoes an electrophysiological study or right atrial ablation. The value of the automated RA LVA is that it can be used as a marker for LA fibrosis, without the need of transseptal catheterization. It seems that the RA mirrors the LA in terms of the extent of low-voltage areas. In line with the literature our results have confirmed the biatrial involvement in atrial fibrillation. An increasing quantity of evidence shows that the extent of LA low-voltage areas can predict the severity of AF and ablation outcomes [29, 30] Therefore, the percentage of RA diseased tissue burden might provide useful information on the severity of AF, which would be a significant potential important clinical implication. However, further studies are still required to confirm the predictive value of the automated RA LVA analysis on the success rate of an AF ablation.

## 5. Limitations

The main limitation is the relatively low number of the involved patients to our single-centre study. The low number of patients with each individual risk factor (especially with diabetes mellitus and prior TIA/stroke) makes this analysis of limited value. Manual exclusion of the appendage, pulmonary veins, mitral annulus, vena cava, and catheter tissue contact may affect the overall accuracy of the HD-EAMs in a minimal degree. Manual registration of each individual point's voltage and subsequent low-voltage area quantification were not part of our protocol, which should be considered as a limitation. The study focused on bipolar voltage alone; however, atrial substrate is thought to be defined by numerous other factors (i.e., conduction velocity, proportion of fractionated points, proportion of double potentials, and conduction/voltage heterogeneity), which has not been investigated in the current study. The new VHA tool has not yet been released in the commercially available CARTO^®^3 electroanatomical mapping systems; thus, the use of the software is currently limited. The present study was not designed to evaluate the RA LVA as a risk factor of AF recurrence after ablation, and a larger scale study would be required to show its clinical impact in that regard.

## 6. Conclusions

Though the treatment and research of AF is focused on the LA, it seems that biatrial remodelling and fibrosis are observed in patients with atrial fibrillation. The VHA tool allows rapid and high definition analysis and determination of the low-voltage areas in the RA. The RA diseased tissue burden shows good correlation with LA diseased tissue burden. Thus, the RA diseased tissue burden might be a useful predictor of the progression of AF; however, this has to be validated by further studies. Our study confirmed previous observations suggesting that the right atrium is a window of the left atrium.

## Figures and Tables

**Table 1:** Baseline patient characteristics.

Baseline characteristics (*n* = 36)		
Gender (female)	9	(25%)
Median age (years)	69	(58–75)
BMI (kg/m^2^)	27	(26–29)
BSA (m^2^)	1.99	(1.78–2.08)
Persistent AF	21	(58%)
CHA_2_DS_2_-VASc ≥2	23	(64%)
LVEF (%)	56	(52–61)
Hypertension	15	(42%)
Diabetes mellitus	4	(11%)
Prior stroke or TIA	3	(8%)
Vascular disease	5	(14%)
Coronary artery disease	8	(22%)
Structural heart disease	9	(25%)
LAVI (CTA) (ml/m^2^)	59	(50–78)
RAVI (CTA) (ml/m^2^)	53	(37–66)

BMI = body mass index, BSA = body surface area, AF = atrial fibrillation, LVEF = left ventricular ejection fraction, TIA = transient ischemic attack, LAVI/RAVI = left/right atrial volume index, and CTA = CT angiogram.

**Table 2:** Procedural characteristics.

Procedural characteristics (*n* = 36)		
Number of LA points	987	(680–1165)
Number of RA points	897	(658–1153)
LA mapping time (min)	8	(6–9)
RA mapping time (min)	7	(6–8)
Diseased LA tissue (%)	19	(13–53)
Diseased RA tissue (%)	24	(14–34)
LAVI (HD-EAM) (ml/m^2^)	78	(60–84)
RAVI (HD-EAM) (ml/m^2^)	87	(71–100)

LA/RA = left/right atrium, LAVI/RAVI = left/right atrial volume index, and HD-EAM = high-density electroanatomic map.

**Table 3:** Relationship between baseline characteristics and percentage of diseased RA tissue burden (≤0.5 mV) with *p* values calculated by the Mann–Whitney *U* test.

	Parameter present		Parameter absent		*p* value
	Number of patients	Diseased RA tissue burden (median)	Number of patients	Diseased RA tissue burden (median)	
Female gender	9	30%	27	21%	0.1796
Age ≥ 65	20	30%	16	20%	0.1222
BMI ≥ 30 kg/m^2^	6	27%	30	23%	0.7877
BSA ≥ 2 m^2^	16	22%	20	24%	0.7089
Persistent AF	21	28%	15	19%	0.0712
CHA_2_DS_2_-VASc ≥ 2	23	30%	13	15%	0.0305
LVEF < 50%	4	26%	32	24	0.9805
Hypertension	15	21%	21	28%	0.9686
Diabetes mellitus	4	27%	32	21%	0.2916
Previous stroke or TIA	3	24%	33	24%	0.7459
Vascular disease	5	30%	31	21%	0.3476
Coronary artery disease	8	27%	28	23%	0.5369
LAVI (CTA) ≥ 59 ml/m^2^	17	30%	18	15%	0.0223
RAVI (CTA) ≥ 53 ml/m^2^	17	30%	18	20%	0.1094
LAVI (HD-EAM) ≥ 78 ml/m^2^	18	31%	18	16%	0.0064
RAVI (HD-EAM) ≥ 87 ml/m^2^	18	31%	18	15%	0.0026
LA-Q4	9	38%	27	19%	0.0007

BMI = body mass index, BSA = body surface area, AF = atrial fibrillation, LVEF = left ventricular ejection fraction, TIA = transient ischemic attack, LA/RA = left/right atrium, LAVI/RAVI = left/right atrial volume index, CTA = CT angiogram, and HD-EAM = high-density electroanatomic map.

## Data Availability

All data used to support this study are available from the corresponding author upon request.
